# Gingival Recession in Primary Maxillary Incisors due to Fingernail Scratching: A Rare Case Report

**DOI:** 10.1155/2024/2278083

**Published:** 2024-07-27

**Authors:** Dalal Maalem, Jalila Hammouti, Hind Ramdi

**Affiliations:** Pedodontics and Preventive Dentistry Department Faculty of Dentistry University Mohammed V of Rabat, Rabat, Morocco

**Keywords:** case report, fingernail-scratching habits, gingival injuries, gingival recession, self-inflicted

## Abstract

**Introduction:** Self-inflicted gingival injuries typically occur in patients with psychological disorders and rarely in normal individuals. This article is aimed at reporting an unusual case of gingival recession caused by a fingernail-scratching habit.

**Case Report:** A 5-year-old female patient presented to the Pediatric Dentistry Department at the Ibn Sina Center for Consultation and Dental Treatment in Rabat, Morocco, with complaints of gingival recession localized in her four maxillary primary incisors. After a detailed medical and personal history and clinical examination, the diagnosis of self-inflicted gingival injury was established. The treatment plan included oral hygiene instructions, the application of analgesic and antiseptic gel, and behavioral management. Regular follow-ups over a period of 16 months were crucial for monitoring the patient's progress, which eventually led to the cessation of the habit with no recurrence.

**Discussion:** Self-inflicted oral injuries in pediatric patients pose diagnostic challenges. The development of such habits in psychologically normal children is difficult to explain, suggesting the need for a comprehensive approach. Managing self-inflicted injuries is complex and requires a personalized strategy that may include psychotherapy, family support, and regular monitoring.

**Conclusion:** This case highlights the importance of taking a comprehensive history and adopting a multidisciplinary approach to diagnose and manage self-inflicted gingival injury, achieving positive outcomes.

## 1. Introduction

Self-inflicted gingival injury, also referred to as “gingivitis artefacta” or “factitial gingivitis,” is a specific type of physical trauma to the gingival tissues [1, 2]. These injuries can be accidental, premeditated, or the result of unconscious habits such as sucking digits or objects (pens, pencils, pacifiers...), fingernail biting, rubbing, scratching, or picking the gingiva using fingernails or abrasive objects, leading to gingival swelling, ulceration, abscess formation, and gingival recession [3–5].

Self-inflicted oral injuries occur more frequently in children than in teenagers and adults, with most reported cases involving female patients [3, 6].

Stewart and Kernohan [7] have distinguished three types of self-inflicted gingival injuries in patients of normal intelligence based on the following etiology:

– Type A: injuries occurring upon an existing lesion or irritation
– Type B: injuries resulting from another established habit like nail biting or finger sucking
– Type C: injuries with uncertain or complex causes often linked to emotional disturbances or psychological illness

Stewart [8] described two variants of gingivitis artefacta: minor and major. Gingivitis artefacta minor, the more prevalent but less serious form, is characterized by superficial gingival lesions caused by repeated rubbing or picking with fingernails or sharp objects. Gingivitis artefacta major, on the other hand, is a more severe form with extensive lesions, often associated with a deeply entrenched habit.

This report presents an unusual case of gingival recession due to a fingernail-scratching habit in a 5-year-old girl.

## 2. Case Report

A 5-year-old girl, accompanied by her mother, reported to the Pedodontics and Preventive Dentistry Department at the Ibn Sina Center for Consultation and Dental Treatment in Rabat, Morocco, with a complaint of gingival recession localized to the four maxillary primary incisors. Her medical history did not reveal any abnormalities.

**Figure 1:**
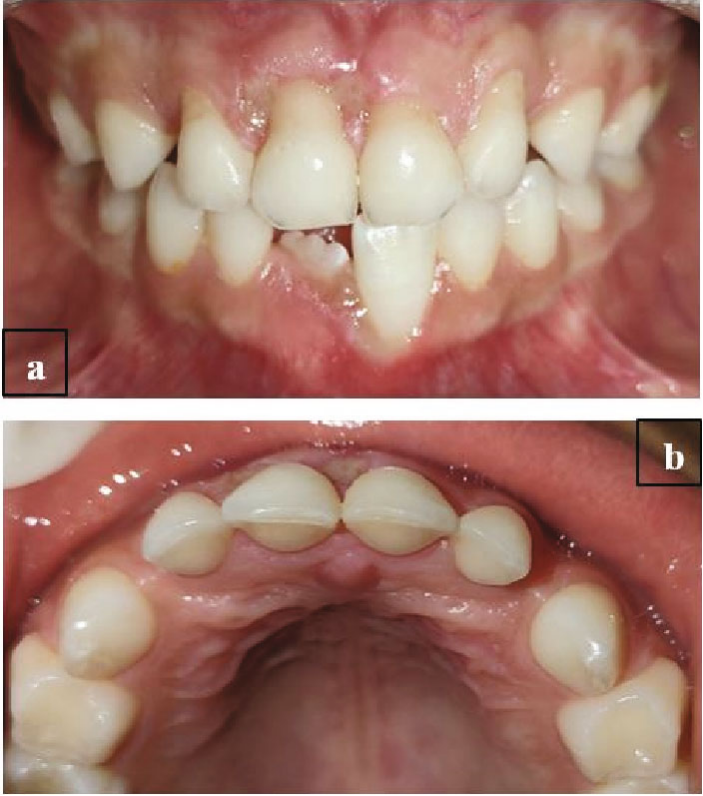
(a) Frontal view of the initial clinical aspect showing the self-inflicted gingival recession with an ulcerated margin on the buccal gingiva of the maxillary incisors, caused by fingernail scratching of the gingiva. (b) Maxillary occlusal view showing no evidence of clinical alterations in the palatal gingiva of the maxillary incisors.

**Figure 2:**
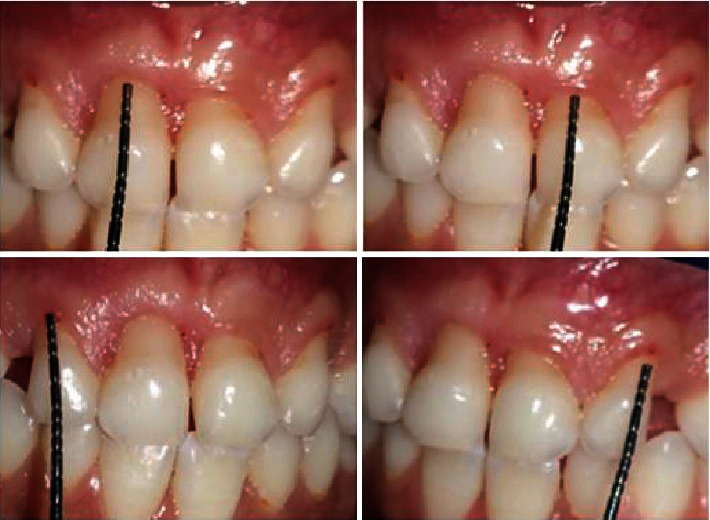
Measuring gingival recession height on the four maxillary incisors.

**Figure 3:**
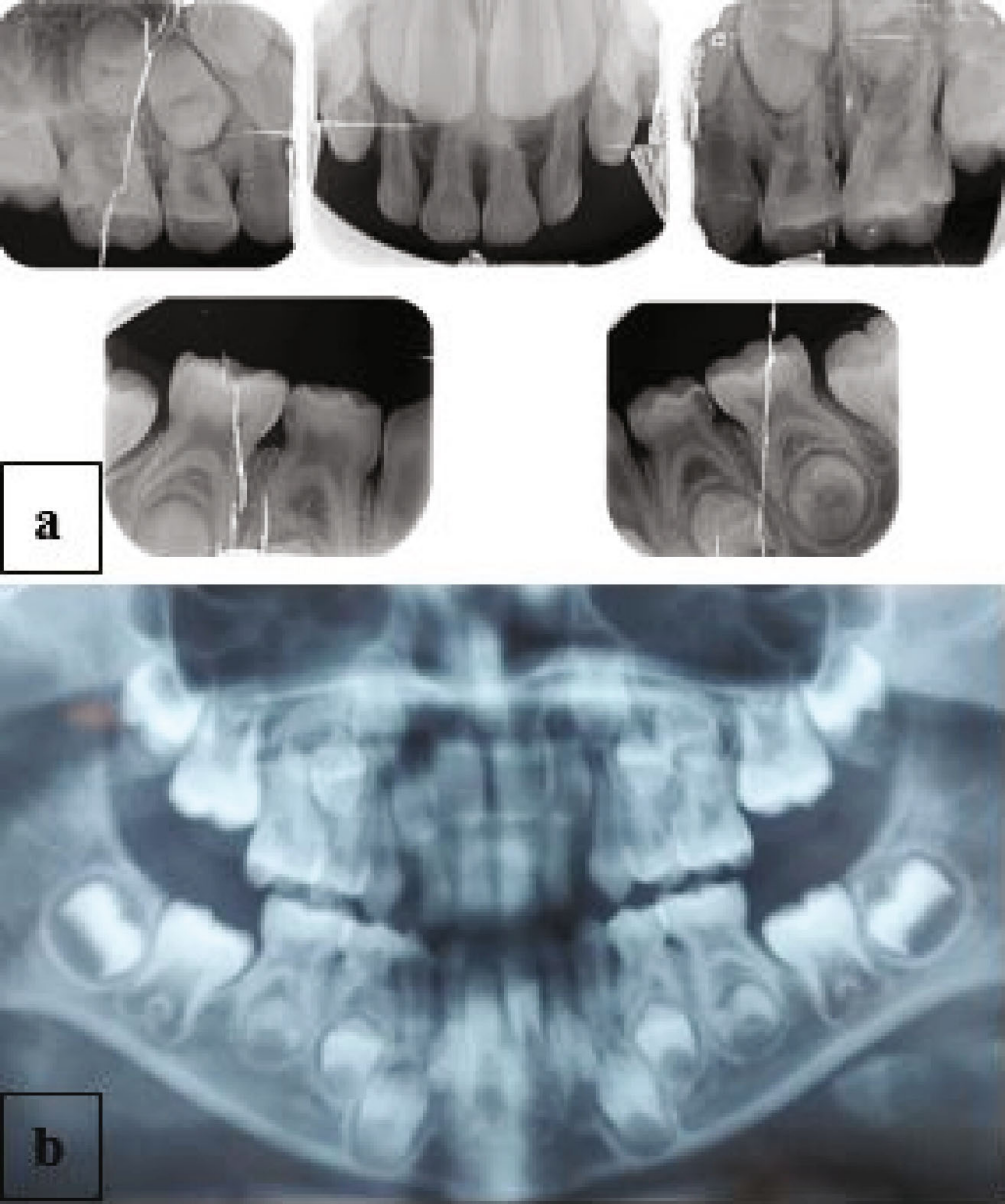
(a) Intraoral periapical and (b) panoramic radiographs showing interproximal bone loss in the incisors and normal alveolar bone in the molars of the primary teeth.

The intraoral examination revealed a complete primary dentition, minimal plaque deposits, and gingival recession on the buccal surfaces of the four upper primary incisors (Figure 1), extending 2 to 5 mm apical to the cementoenamel junction without papilla loss (Figure 2). The incisors showed no mobility, and the gingiva in these regions appeared ulcerated. There was no bleeding on probing, and there were no periodontal pockets. A periapical radiograph showed interproximal bone loss in the incisors, with no abnormalities detected in the molars (Figure 3).

Upon questioning the mother about any unusual practices or habits related to the child's oral health, she reported that the child had been scratching her gums with her fingernails in the affected area for the past 2 years. A comprehensive family history assessment was undertaken to exclude the possibility of any negative familial background. All results were negative, and there was no evidence of previous emotional disturbances in the child's history.

**Figure 4:**
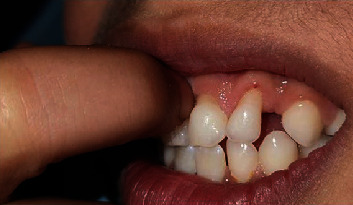
Patient demonstrating her fingernail-scratching habit on the gingiva.

The patient reported sensitivity and irritation in the affected area, which led to gum scratching. To rule out any underlying systemic conditions, a complete blood count, alkaline phosphatase levels, creatinine levels, T4 lymphocyte counts, and coagulation factors were analyzed and found to be normal. We concluded that the observed gingival recession was primarily due to self-gingival injury. The diagnosis was confirmed by observing the finger placement in the specific area after inquiring about the patient's technique (Figure 4).

The treatment approach emphasized oral hygiene instructions, the prescription of a mouth gel with analgesic and antiseptic properties, and behavior management. Effective communication with the patient and her mother was essential to raise awareness regarding the harmful effects of this habit on gingival health. Moreover, the parents were informed about the importance of their active involvement in monitoring the child's behavior and providing encouragement and a stress-free environment to achieve successful outcomes.

Frequent follow-up appointments were crucial for monitoring the child's progress. The patient was seen monthly for the first 3 months, followed by regular check-ups every two to 3 months. These visits focused on assessing the recession depth, monitoring behavior, and ensuring continued oral health.

**Figure 5:**
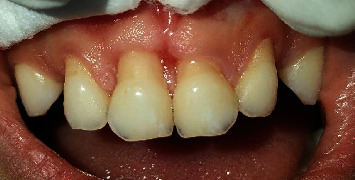
Clinical view after 2 weeks: initial healing of the ulcers.

**Figure 6:**
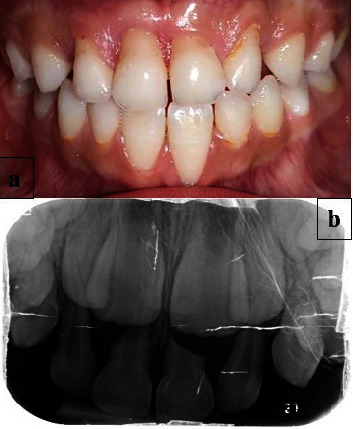
Follow-up of the patient after 6 months: (a) clinical view showing no progression of the gingival recession and (b) periapical radiograph showing physiological root resorption of central incisors.

At the 15-day follow-up (Figure 5), initial healing of the ulcers was observed. By the 3-month follow-up, the gingival recession remained stable, showing no further progression. The child demonstrated a significant improvement in controlling her habit, leading to reduced discomfort and irritation. Notably, the patient exhibited complete cessation of her habit by the 6-month follow-up period (Figure 6).

**Figure 7:**
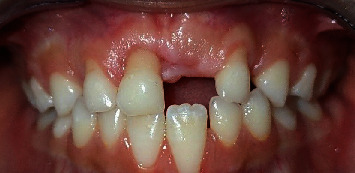
Follow-up after 8 months: physiological shedding of the left maxillary central incisor.

**Figure 8:**
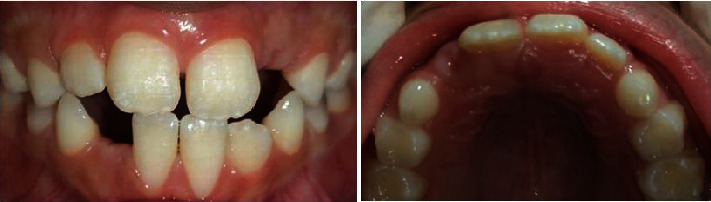
Clinical view after 16 months showing healthy gingival tissue surrounding the erupted permanent incisors, with no recurrence of the fingernail-scratching habit of the gingiva.

Throughout the following 16 months, during which her primary incisors were replaced by permanent teeth, the child showed no recurrence of the gingival scratching habit or any similar behavior (Figures 7 and 8). This positive change contributed to the healthy eruption of her permanent incisors, confirming the success of the comprehensive treatment approach.

## 3. Discussion

Self-inflicted oral injuries present diagnostic challenges for pediatric dentists, requiring a comprehensive medical history and thorough clinical examinations for accurate diagnosis and effective treatment [9]. These injuries can be classified as either organic or functional. Organic self-mutilation is commonly associated with genetic syndromes, medical conditions, and neurological or psychiatric disorders, such as Gilles de la Tourette syndrome, Lesch-Nyhan syndrome, Cornelia de Lange syndrome, congenital insensitivity to pain, autism, and intellectual disabilities [10]. In these cases, the injuries are inflicted unconsciously and compulsively, without specific intent. Conversely, functional self-mutilation involves deliberate actions, often in response to certain stimuli or the need for attention [11].

According to Pattison [3], it remains challenging to explain why a child with normal psychological behavior and intelligence would develop such a habit. However, it is possible that underlying minor stressors or transient psychological factors could play a role. The motivations for self-inflicted behavior can range from seeking attention or obtaining desired materials to avoiding stressful situations or relieving psychic pain [12].

Stewart and Kernohan [7] identified the following several characteristics typical of self-inflicted injuries to the gingiva:

– They do not correspond to any recognized pathological entity.
– They exhibit an unusual configuration with sharp outlines on an otherwise normal background.
– The distribution of these lesions is often atypical and frequently occurs in areas accessible to the patient's hand.
– They may occur singly, but more often, they are multiple.

There are varying degrees of self-injurious behavior, ranging from mild fingernail biting or scratching the gingiva to severe self-mutilation such as tooth extraction or biting the lip and tongue, leading to significant destruction. However, self-inflicted injuries affecting these sites have been reported particularly in patients with a genetic syndrome [3, 12].

In this case, the lesions in the patient were characterized by gingival recession and did not correspond to any known disease. The lesions exhibit an unconventional configuration, were bilateral, and were easily accessible to the patient's hand, as described by Stewart and Kernohan [7]. Furthermore, the patient reported regular scratching of the gingival area, which, combined with the clinical lesion manifestation and the exclusion of plaque-induced or systemic periodontitis, suggests self-inflicted gingival injury.

Management of self-inflicted injuries can be complex due to the lack of established strategies for preventing or treating orofacial self-inflicted injuries. It may be difficult for the patients to stop their harmful habits. The treatment plan will depend on the patient's specific circumstances and may involve a multidisciplinary approach, including local/preventive measures, referral to psychotherapy in cases of emotional disturbance, and periodic clinical control of recurrences [5, 6, 13].

Oral appliance therapy is used in the literature as a physical restraint method to discourage patients from performing certain habits. These appliances help suppress the offending behavior and prevent traumatization of oral tissues [10, 11]. While these devices may effectively suppress oral self-inflicted behavior, failure to address the underlying reasons for the behavior could result in the emergence of alternative forms of self-injurious behavior [14].

Various behavioral interventions have shown efficacy in addressing self-injurious behaviors, such as positive reinforcement using attention or tangible rewards, negative reinforcement through task escape or avoidance, and sensory reinforcement. These interventions are aimed at replacing harmful habits with more adaptive behaviors. They investigate the root causes by understanding the patient's specific needs and communication challenges [11, 14, 15]. Additionally, the active involvement of parents is crucial. The dedication of parents to closely monitoring their child, providing encouragement, and addressing the harmful habit plays a significant role in the successful treatment outcome.

Consistent follow-up, active social support, and ongoing communication with patients and their families are essential. These elements enable healthcare providers to address and effectively prevent the recurrence of these harmful behaviors [16].

## 4. Conclusion

This case highlights the importance of considering self-inflicted gingival injuries, such as fingernail scratching habits, as potential causes of gingival problems. It also reinforces the value of early intervention, behavior modification, and consistent follow-up in achieving successful treatment outcomes in pediatric dentistry.

## Data Availability

The data (figures) supporting the findings of this case report are included within the manuscript and are available upon request.

## References

[1] Rajiv S., Biju T., Maithreyi V. P. (2010). Self-inflicted traumatic injuries of the gingiva- a case series. *Journal of International Oral Health*.

[2] Chapple I. L., Mealey B. L., Van Dyke T. E. (2018). Periodontal health and gingival diseases and conditions on an intact and a reduced periodontium: consensus report of workgroup 1 of the 2017 world workshop on the classification of periodontal and peri-implant diseases and conditions. *Journal of Periodontology*.

[3] Pattison G. L. (1983). Self-inflicted gingival injuries: literature review and case report. *Journal of Periodontology*.

[4] Blanton P. L., Hurt W. C., Largent M. D. (1977). Oral factitious injuries. *Journal of Periodontology*.

[5] Shah N., Dave B. (2014). Factitious gingival habit: a case report. *Scholars Journal of Applied Medical Sciences (SJAMS)*.

[6] Dilsiz A., Aydin T. (2009). Self-inflicted gingival injury due to habitual fingernail scratching: a case report with a 1-year follow up. *European Journal of Dentistry*.

[7] Stewart D. J., Kernohan D. C. (1972). Self-inflicted gingival injuries. Gingivitis artefacta, factitial gingivitis. *The Dental Practitioner and Dental Record*.

[8] Stewart D. J. (1976). Minor self-inflicted injuries to the gingivae. *Journal of Clinical Periodontology*.

[9] Mustafa M. M. E., Almosa A. A. M., Alshahrani A. M. S., Alshahrani S. M. (2016). Gingivitis artefacta minor secondary to poor endodontic treatment of a primary molar. A case report. *International Journal of Medical and Dental Case Reports*.

[10] Galeotti A., Aristei F., Putrino A. (2024). Oral self-inflicted accidental trauma in patients with neurological disorders: a case report of dental management in infants with cerebellar hypoplasia. *Journal of Clinical Pediatric Dentistry*.

[11] Bhattarajee S., Iyer S. S., Panigrahi A., Mohanty S., Mohanty B. (2018). Oral self injurious habits-a review. *Indian Journal of Public Health Research & Development*.

[12] Hildebrand L. C., Carvalho A. L., da Rosa F. M., Martins M. D., Filho M. S. (2011). Functional oral self-mutilation in physically healthy pediatric patients: case report and analysis of 27 literature cases. *International Journal of Pediatric Otorhinolaryngology*.

[13] Çalışkan S., Tüloglu N., Biçer H., Bayrak S. (2018). Oral self-mutilation: two case reports. *Clinical Dentistry and Research*.

[14] Romer M., Dougherty N. J. (2009). Oral self-injurious behaviors in patients with developmental disabilities. *Dental Clinics of North America*.

[15] Pattnaik N., Satpathy A., Mohanty R., Nayak R., Sahoo S. (2015). Interdisciplinary management of gingivitis artefacta major: a case series. *Case Reports in Dentistry*.

[16] Stoufi D. E., Georgaki M., Tsouri I., Nikitakis N. G. (2021). Self-inflicted oral mucosal injuries in pediatric patients: a case series. *Interventions in Pediatric Dentistry: Open Access Journal*.

